# Chemical interaction between sea-salt and tellurium, between 300 and 1180 K

**DOI:** 10.1007/s10967-018-5922-1

**Published:** 2018-05-30

**Authors:** Fredrik Espegren, Henrik Glänneskog, Mark R. StJ. Foreman, Christian Ekberg

**Affiliations:** 1 Department of Chemistry and Chemical Engineering, Nuclear Chemistry and Industrial Materials Recycling, Chalmers University of Technology, Kemivägen 4, 412 96 Göteborg, Sweden; 2 Vattenfall, AB, 401 27 Göteborg, Sweden

**Keywords:** Tellurium, Sodium chloride, Thermogravimetric, Fukushima

## Abstract

As an emergency action during the Fukushima accident, seawater was used to maintain cooling. To evaluate the effect of the salt on fission-products, sodium chloride, and tellurium were heated together using different ratios in different atmospheres (inert or oxidizing) using thermogravimetric analysis. The experiment under inert conditions showed no indication of interaction. However, under oxidizing conditions an interaction for all samples was observed that prevented an otherwise observed mass increase of the tellurium reference. The change in the appearance of the samples at increasing temperatures was studied by heating them in a furnace.

## Introduction

During the Fukushima Daiichi accident, the loss of plant cooling of several reactor units resulted in the need for emergency cooling using seawater [1, 2]. Utilizing seawater as a coolant creates a potential chemical issue since the salt concentration in seawater is high (chloride content 18.980 mg/l) [3]. Thus, a chemical reaction could enhance the source term of otherwise less volatile fission-products through the formation of new more volatile, chloride species.

The literature regarding research on the chemical effects of using seawater as an emergency cooling of fission-products is scarce. It is known that many chlorides of different fission-products have lower boiling points than that of the pure metallic state [4], thus requiring less heat before volatilizing and subsequently being released from the fuel.

Moreover, it has been reported that unexpected behavior of a long-lived fission product has been observed in the air 175 km southwest of Fukushima, at Tsukuba [5]. Instead of following the model prediction based on expected physical decay, the long-lived fission product activity has increased over time. One of the explanations of this behavior proposed by the author was the formation of fission product halides [5]. This is possible, as the chlorides of many fissions products have a lower boiling point than the metallic state. One example of the significant change in boiling points is niobium, where the boiling point drops from 5031 K (metallic) to 520 K (NbCl_5_) [4].

This could also be the case for other more volatile fission-products as well. A likely candidate would be tellurium, as its halide compounds (e.g. Te_4_Cl_16_, 663 K [4]) have lower boiling points than the metallic state (1263 K [4]). Chemically tellurium is also a highly reactive element [6] and releases of different tellurium isotopes contributed considerably to the total activity releases during the Fukushima Daiichi accident, as reported by Le Petit et al. [7]. These arguments imply that the use of seawater as emergency cooling enhanced the source term of tellurium.

Experiments were therefore designed and carried out to determine the interaction of sodium chloride with tellurium under two atmospheres, inert and oxidizing. The initial experiments were made using thermogravimetric analysis (TGA) to determine if a reaction occurs between tellurium and sodium chloride. To gain a visual overview of the sample at selected temperatures, heating in a furnace was carried out under oxidizing conditions.

## Tellurium chemistry

The extent of tellurium relevance from a radiological standpoint is considerable. The most abundant isotopes of tellurium in the fuel are ^131^Te, ^132^Te, ^133m^Te and Te^134^, which constitutes 60% [6] of the total amount of tellurium. Moreover, fission product content at the end-of-life for a 900 MWe pressure water reactor has been calculated elsewhere [8] by using the DARWIN-Pepin code. The calculation was based on a core contain 72.5 × 10^3^ kg uranium, divided into four quarters, each with an individual burnup of 10.5, 21, 31.5 and 42 GWd/t. The results from these calculations showed that the amount of total tellurium was 26.2 kg, which can be compared to the iodine content of 12.7 kg and cesium content of 161 kg.

An important behavior that is specific to tellurium is that tellurium is trapped by zircaloy cladding [9–11]. Tellurium reacts with the zirconium present in the cladding to form zirconium tellurides. Therefore, tellurium becomes trapped and releases of tellurium from the core are delayed.

For any trapped tellurium to be released from the cladding, the cladding needs to be sufficiently oxidized [10]. The oxidation occurs when the cladding no longer is covered by water and exposed to steam [12], according to

1 $${\text{Zr}} + 2{\text{H}}_{2} {\text{O}} \to 2{\text{H}}_{2} + {\text{ZrO}}_{2} + Q_{Heat}$$

Considering that tellurium is trapped in the cladding, a potential dryout after using seawater for cooling could enable the chemical reaction between tellurium and salt on the cladding surface. Moreover, ballooning and burst of the cladding when temperature rises and external pressure around the cladding is lost [12], would make it possible for seawater to reach the fuel pellet itself. This would enable reaction on the surface of the fuel pellet.

## Experimental

### Thermogravimetric analysis

The main parts constituting the samples were metallic powder tellurium (99.8%, 200 mesh, Sigma Aldric) and sodium chloride (99.5%, Acros Organics). The latter was ground down using a mortar and pestle to attain a finer powder. The aim was to improve the mixing of tellurium and sodium chloride by having similarly sized powders.

Three samples (S1, S2, and S3) and two references (Ref1 and Ref2) were investigated. The ratios for each sample investigated were 4:1, 1:1 and 1:4 of tellurium and sodium chloride. The references were made from the same materials, but with only one of the compounds used. Triplicates were made of all samples and references.

Every sample (7–9 mg) was placed in an alumina pan (100 μl, TA Instrument), which had been cleaned with 1 M HNO_3_ (made from 70% ACS reagent, Sigma Aldrich) and Milli-Q water (Millipore, 18 MΩ) prior to use and then left to dry in a drying cabinet overnight. After adding the sample, it was then spread out in the pan by gently tapping one side of the pan before placing the pan and sample inside the TGA (TGAQ500, TA Instrument).

A gas flow of 90 ml/min was maintained throughout the whole experiment and was used to establish the desired atmosphere, which for inert conditions was nitrogen (99.98%, in-house gas) and for oxidizing conditions was synthetic air (79/21% nitrogen/oxygen, AGA). A second flow of nitrogen was also used to support the pan (10 ml/min, 99.98%, in-house gas). No external monitoring of the flow was done. Instead, all gas flows were monitored by the TGA-equipment itself.

Heating (5 K/min) was performed from ambient temperature to isothermal temperature (1173 K, max temperature of the TGA) for both atmospheres. Heating was maintained for 20 min at isothermal temperature, after which the system was allowed to cool to ambient temperature. All temperatures were monitored by the equipment, without external verification. The measurement was performed by the TGA with a one second interval, between each measurement point.

All outgoing pipes from the TGA were cleaned with acetone after each run. This was followed by an empty run under inert conditions and a final cleaning of the pipes with acetone.

### Tubular furnace experiments

**Fig. 1:**
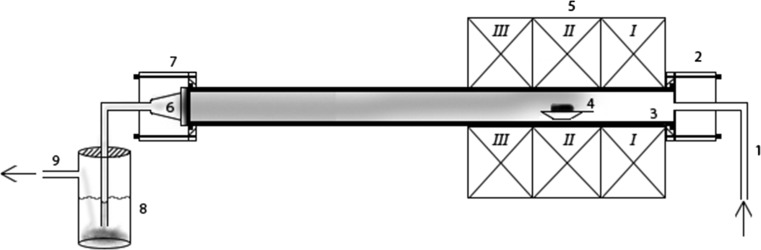
The experimental setup used: (1) gas flow inlet, (2) cylindrical formed inlet connector made from stainless steel, (3) ceramic tube of length 130 cm, (4) the mixture of compounds, (5) tubular furnace (6) filter holder, (7) cone formed outlet connector made from stainless steel, (8) cold trap used to prevent particles escaping, and (9) gas flow outlet. The tubular furnace is divided into three heat zones: in Heat Zone I the gas flow is heated to the programed temperature, in Heat Zone II the gas flow maintains at least the programed temperature, and in Heat Zone III the temperature of the gas flow cannot be maintained and thus decreases

To attain a visual observation of the sample after heating to several different temperatures, experiments were performed inside a tube furnace (ETF 30-50-18-S, Entech). A 130 cm high purity alumina tube (Al_2_O_3_, 99.7%, Degussit AL23, Aliaxis) was used inside the furnace. Connectors (stainless steel, custom made) were added to both ends of the tube. To promote a smoother transition of the gas flow at the outlet, the connector here was designed with an internal shape of a cone. An overview of the experimental setup used can be seen in Fig. 1.

The sample ratio investigated was 1:1 using the same supplier and quality for tellurium and sodium chloride (no grinding), but with a total weight of 2 g. Each sample was placed inside a crucible (Boat, Porcelain 85 × 13 × 8 mm, VWR) before being placed inside the alumina tube.

The experiments in the furnace were performed under oxidizing conditions, where a gas flow (1.5 l/min, Aalborg gas regulator) of synthetic air (compressed air) was used. Moreover, to ensure oxygen in excess the gas flow was maintained throughout the whole experiment.

Rapid heating (10 K/min) was carried out from ambient temperature up to isothermal temperature (473 K and several temperatures in the interval 573–1073 K). This in order to reach isothermal temperature as soon as possible. The isothermal temperature was maintained for 20 min. Following this, the system was left to cool to room temperature under the gas flow (1.5 l/min) used to establish the atmosphere.

## Results and discussion

**Fig. 2:**
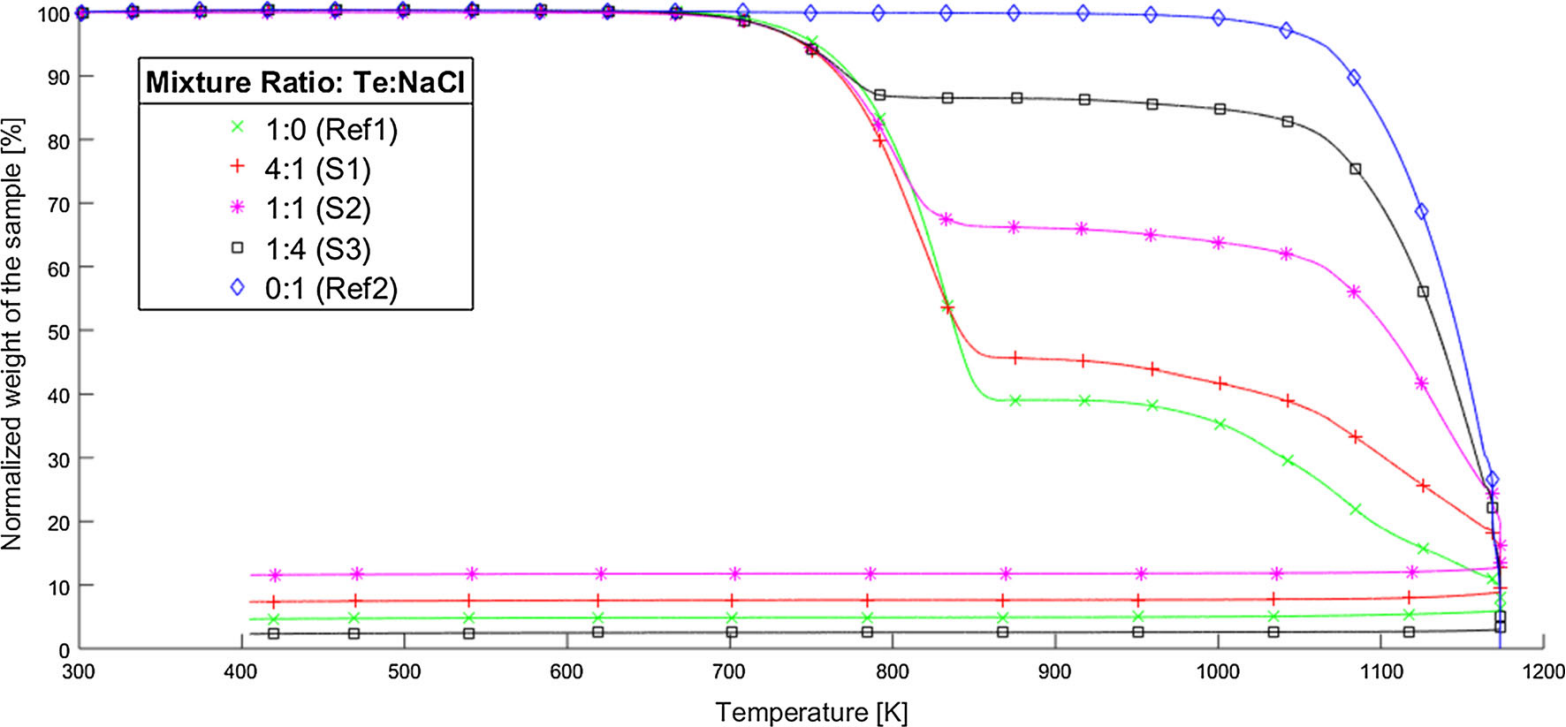
The thermogravimetric analysis results for the different Te:NaCl-ratios (weight basis) heated in inert conditions. The different lines represent the average of three replicates of 1:0 (Ref1, green crosses), 4:1 (S1, red pluses), 1:1 (S2, pink stars), 1:4 (S3, black boxes), and 0:1 (Ref2, blue diamonds) of tellurium and sodium chloride respectively. All weights have been normalized towards the first measured weight (8–9 mg) by thermogravimetric analysis. The measuring interval was one second. (Color figure online)

**Fig. 3:**
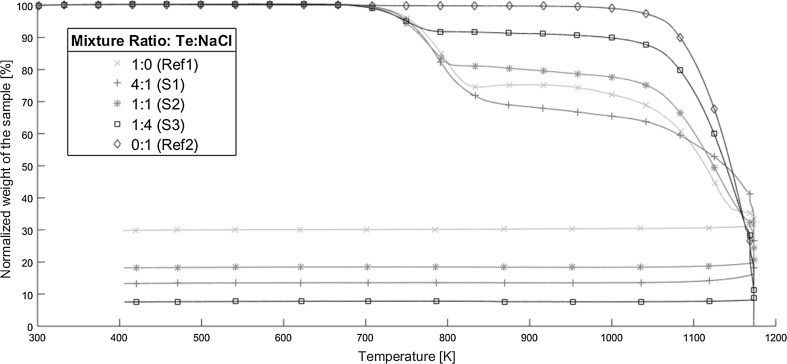
The thermogravimetric analysis results for the different Te:NaCl-ratios (weight basis) heated in oxidizing conditions. The different lines represent the average of three replicates 1:0 (Ref1, green crosses), 4:1 (S1, red pluses), 1:1 (S2, pink stars), 1:4 (S3, black boxes), and 0:1 (Ref2, blue diamonds) of tellurium and sodium chloride respectively. All weights have been normalized towards the first measured weight (8–9 mg) by thermogravimetric analysis. The measuring interval was one second. (Color figure online)

From the TGA experiments, a normalized mass-loss as a function of temperature is produced. The results can be seen in Fig. 2 for inert conditions and in Fig. 3 for oxidizing conditions. In these two figures, five curves can be seen, each either representing one of the mixture samples (S1 red pluses, S2 pink stars, and S3 black squares), or the references (Ref1 green crosses and Ref2 blue diamonds).

### TGA, inert conditions

Beginning with the tellurium heated alone under inert conditions in Fig. 2 (Ref1, green), the mass loss remains unchanged until 690–715 K. At these temperatures, a mass loss begins and continues until 855–870 K, where it stops, and the change becomes almost flat. First, at 920–960 K, a new mass loss is observed. This mass loss remains until the end of the experiment and after the cooling (the final straight line), the final normalized mass ends at 5% of the initial value.

The result for when sodium chloride alone was heated under inert conditions, can be seen in Fig. 2 (Ref2, blue). For sodium chloride, only one mass loss can be observed. This starts at 1000–1075 K and is maintained until the end of the experimental run, including the cooling part. At that, point almost no mass is left.

The first sample (S1, red) in Fig. 2 when heating under inert conditions is a mixture consisting mainly of tellurium and to a lesser extent sodium chloride. No change to the mass is observed until temperatures of 680–720 K are reached, then the first mass loss begins. This mass loss remains until temperatures of 850–865 K, where the mass change halts and the curve almost completely flatten out. Another mass loss does not occur until 925–950 K. This mass loss continues until the end of the experiments and the final mass ends at 8% of the original amount after the cooling (the final straight line).

For the sample consisting of equal amounts of tellurium and sodium chloride, the result of heating in inert conditions is shown in Fig. 2 (S2, pink). The mass of the sample remains unchanged until temperatures of 680–725 K are reached, where a mass loss begins. This mass loss is maintained until 830–850 K, after that it is significantly reduced and starts to flatten out. A significant mass loss does not occur again until the temperature reaches 920–945 K. The mass loss remains until the end of the experiment, ending after cooling (the final straight line) at a normalized mass of 12% compared to the first value.

The final sample heated under inert conditions corresponds to when sodium chloride constitutes the major part and tellurium the minor part of the sample (S3). The result can be seen in Fig. 2 (S3, black). This sample remains unchanged until the temperatures reaches 670–700 K, where the first mass loss occurs. The mass loss remains until the temperature reaches 785–805 K, where it almost stops and remains unchanged until 920–960 K. At these temperatures, the final mass loss occurs and continues until the end of the experiment. The final normalized mass after cooling (the final straight line) is 3% compared to the original mass.

In Table 1, an overview of the experiment carried out under inert conditions can be seen. It contains the temperature for the two main mass losses and the final normalized mass after the cooling.

**Table 1:** A summary of the thermogravimetrically analysis carried out under inert conditions. Each value seen is the smallest value for the temperature interval for the start of the mass loss and the largest value of the temperature interval for when it ends. The final normalized mass is after the cooling

Sample	First main mass loss temperature range (K)	Second main mass loss temperature range (K)	Final normalized mass (wt%)
Ref1	690–870	920 to end	~ 5
Ref2	1000 to end	–	~ 0
S1	620–865	925 to end	~ 8
S2	680–850	920 to end	~ 2
S3	670–805	920 to end	~ 3

The first mass losses that occur for S1, S2 and S3 are most likely similar to what is happening during the first mass loss of the tellurium reference (Ref1). Considering the temperatures for when the mass losses occur, these are close to the melting point of tellurium (725 K [4]) and, as such, a phase change from solid to liquid is most likely occurring. Concurrently with the phase change of tellurium, something is also being volatilized. As the reference also shows losses at these temperatures, tellurium vapor would be a likely candidate. According to the literature, the volatilized species would be either Te_2_ or Te as these are expected to be the gaseous species of tellurium [13]. The observed mass loss of the different samples ends at different normalized masses after cooling (e.g. at the final straight line of each sample). Where the samples S2 remains at the end with the highest mass loss, followed by S1 and then S3.

After the initial mass loss, the change stops and becomes almost zero. This lack of change occurs at different temperature intervals for all samples. This observation can be attributed to the presence of sodium chloride. As the temperature needed before a mass loss occurs again increases with the sodium chloride content.

The final mass losses that occur for the samples are all at different temperatures and ends at different normalized masses. Again, the content of sodium chloride is the main difference between the samples. What is happening during these mass losses would be partly volatilization of tellurium (based on the tellurium reference), but also the melting and phase transition of sodium chloride (melting point 1074 K [4]). The latter is also being volatilized on its own, as the sodium chloride reference is also undergoing a mass loss at these temperatures.

A general observation is that the mass loss behavior of the different samples in inert conditions is dictated by the main constituting part of the sample, i.e. the amount of tellurium or sodium chloride. As experiment S1 highly resembles the Ref1 case, S3 is more similar to Ref2 and S2 is somewhere in between.

Thus, the results in Fig. 2 are indicative that no reaction has occurred between tellurium and sodium chloride for any of the samples. However, as tellurium has a considerably lower melting point than sodium chloride there might not be enough tellurium available when a temperature is reached where the tellurium could be affected by sodium chloride. Thus, it is conceivable that no reaction is observable from the result as the tellurium volatilized would be almost instantly transported away by the gas flow and little to nothing would remain when a potential reaction could occur. Therefore, a reaction could still occur at a higher temperature if tellurium remains.

That tellurium would remain at higher temperature is possible as it is trapped by unoxidized cladding and therefore could remain at a higher temperature. Moreover, tellurium would be released only when the cladding becomes sufficiently oxidized [7, 10, 11].

### TGA, oxidizing conditions

The next set of experiments were carried out under oxidizing conditions. Starting with the references in Fig. 3, the tellurium (Ref1, green) behavior is unchanged up to 700–720 K. At these temperatures, a mass loss begins and goes on until 820–840 K, where the mass loss completely stops and slowly becomes a mass increase. This mass increase is maintained up to temperatures of 900–940 K. Following this increase, a rapid mass loss occurs that lasts until the end of the experiment. The final normalized mass loss after cooling (the final straight line) is 30% of the initial amount.

The sodium chloride reference heated under oxidizing conditions can be seen in Fig. 3 (Ref2, blue). This behaves similarly to the reference of sodium chloride seen in Fig. 2 (Ref2, blue) heated under inert conditions. That is, no mass loss is observable until temperatures of 1000–1050 K are reached. A rapid mass loss follows, which lasts until the end of the experiment. The final mass left, is close to zero.

The first sample is seen in Fig. 3 (S1, red) shows the results from heating tellurium with a smaller amount of sodium chloride present in oxidizing conditions. No change to the mass is observed up to temperatures of 700–720 K. The first mass loss begins at these temperatures, which lasts until 845–870 K. At these temperatures, the mass loss drastically slows down and remains steadily up to 1030–1060 K, where a new mass loss occurs. This final mass loss lasts until the end of the experiment, with a final normalized mass of 14% of the original value after cooling (the final straight line).

The second sample is seen in Fig. 3 (S2, pink) is when tellurium and sodium chloride are in equal amounts and are heated under oxidizing conditions. The mass remains unchanged up until temperatures of 695–715 K, where a mass loss takes place. This mass loss remains until temperatures of 795–815 K, as at these temperatures the rate of the mass loss slows considerably. This reduced mass loss lasts until temperatures of 1020–1050 K, where the mass loss drastically increases again. This final mass loss remains until the end of the experiment and after cooling (the final straight line) the final normalized mass halts at 18% of the original amount.

The results for the final sample heated in oxidizing conditions can be seen in Fig. 3 (S3, black), sodium chloride is the major and tellurium the minor part of the sample. The first change to the mass occurs at 680–720 K and lasts until 770–785 K. At this point, the mass loss slows down and almost becomes zero. The normalized mass remains unchanged up to temperatures of 1000–1030 K, where a final mass loss occurs and continues until the end of the experiment, ending at a normalized mass of 8% of the initial value after the cooling (the final straight line).

In Table 2, an overview of the experiment carried out under oxidizing conditions can be seen. It contains the temperature for the two main mass losses and the final normalized mass after the cooling.

**Table 2:** A summary of the thermogravimetrically analysis carried out under oxidizing conditions. Each value seen is the smallest value for the temperature interval for the start of the mass loss and the largest value of the temperature interval for when it ends. The final normalized mass is after the cooling part

Sample	First main mass loss temperature range (K)	Second main mass loss temperature range (K)	Final normalized mass (wt%)
Ref1	700–840	900 to end	~ 30
Ref2	1000 to end	–	~ 0
S1	700–870	1030 to end	~ 14
S2	695–815	1020 to end	~ 18
S3	680–785	1000 to end	~ 8

During the first mass loss of the tellurium (Ref1), and most likely the three samples (S1, S2, S3) in Fig. 3, a phase change from solid to liquid of the tellurium species is occurring as indicated by the melting point of tellurium (725 K [4]) and that of TeO (643 K [3]). Moreover, the tellurium references mass loss indicates that something is being volatilized. What could be volatilized is either a tellurium vapor species similar to the inert experiment, or a tellurium oxide (e.g. TeO based on the melting point or TeO_2_ considering the phase diagram of Te–O in [14]). A minor difference is occurring between the three samples and the reference of tellurium, as the reference has a slightly slower mass decrease. This difference could be indicative of either a chemical reaction in the samples or that the presence of sodium chloride alters the rate on its own.

After the first mass loss in the tellurium reference, a significant mass increase is observed. This increase can be explained by the formation of α-TeO_2_, as can be observed in the Te–O phase diagram [14]. As a decreasing amount of tellurium would result in a shift towards the vertical line of α-TeO_2_. Moreover, TeO_2_ melts at 1006 K [3] and thus a phase change is taking place at this temperature. The phase diagram of Te–O [14] shows that both a liquid and a gas phase exists above the melting point. Thus, the second mass loss is explained by volatilization of TeO_2_.

However, this increase of mass is only observed for the tellurium reference, as all three samples and the sodium chloride reference do not have any mass increase stage. This is indicative that something has altered the behavior of the tellurium. An explanation could be that the mere presence of sodium chloride could prevent the oxidation of tellurium in the crucible by physically preventing the oxygen from reaching the tellurium. However, another possibility could be that a chemical reaction has occurred between the tellurium and sodium chloride with the result being the formation of a new readily volatilized compound. This new compound would then either surpass the effect of mass increase from the formation of TeO_2_ by volatilization or by reducing the formation of the oxide.

Furthermore, the difference between the three samples are minor, if any, and can be explained by the content of sodium chloride. Increasing the content of sodium chloride results in the sample behaving more similarly to the sodium chloride reference. Thus, this would mean that even small amounts of sodium chloride (e.g. a fifth of the weight of the total sample) would affect the tellurium volatilization.

### Visual inspection, oxidizing conditions

**Fig. 4:**
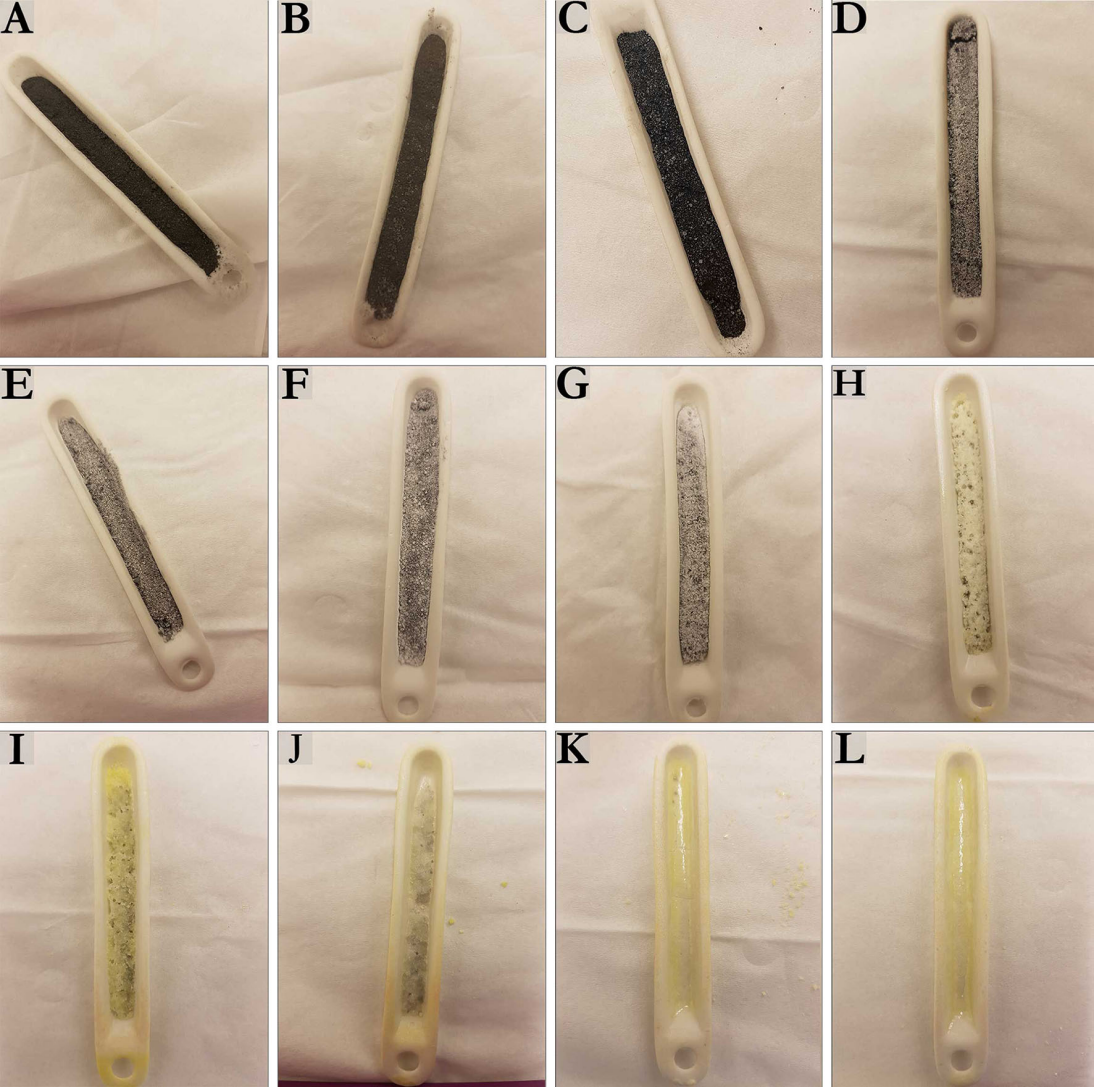
Crucibles containing the sample, as seen after the furnace experiment. The initial composition of the samples was 1:1 of tellurium and sodium chloride (2 g). In total eight, temperatures were investigated: **a** 473 K, **b** 573 K, **c** 623 K, **d** 673 K, **e** 723 K, **f** 773 K, **g** 823 K, **h** 873 K, **i** 923 K, **j** 973 K, **k** 1023 K and **l** 1073 K

To further evaluate the oxidizing conditions, furnace experiments were carried out and the results can be seen in Fig. 4. The initial state of the mixture resembles the crucible in image A; the two parts are tellurium (black powder) and sodium chloride (white powder).

In Fig. 4 several crucibles can be seen, containing what was left after the furnace experiments. Each crucible represents an investigated temperature (473–1073 K) and shows how heating to the temperature affects the sample of 1:1-ratio of tellurium and sodium chloride.

What can be observed is that for the temperatures 473 K (A), 573 K (B) and 623 K (C), the sample remains seemingly unchanged after the experiment. Above 673 K (D) the first observable change can be seen, as white particles (sodium chloride based on the original content of the sample) increase in appearance.

At 723 K (E), the black powder (tellurium based on the original content of the sample) seemingly starts to disappear, which lasts until 873 K (H). At this temperature, the next change can be observed, as a slightly yellow phase starts to emerge that is observable, both on the mixture and on the crucible. Moreover, the sample parts have now merged together and formed a solid mass.

Increasing the temperature to 923 K (I) resulted in the yellow phase becoming even more obvious. The sample has also become even more of solid and the sodium chloride has started to lose its original shape.

At the next temperature, 973 K (J), the mixture has become a solid slug at the bottom of the crucible and occupies a considerable less volume.

Increasing the temperature again to 1023 K (K) drastically changes the appearance as nothing except the yellow phase remains. Some black spots can be observed, but these are most likely tellurium encapsulated in the yellow phase.

The final sample heated to 1073 K (L), show no significant difference from the sample heated to 1023 K.

From the images in Fig. 4, it is possible to see that up to 623 K nothing observable happens to the samples containing tellurium (black particles) and sodium chloride (white particles). This would be consistent with what would be expected based on the melting point of tellurium (725 K [4]). The sample at 673 K shows an increasing number of white particles, the reason for which is not clear based on these images. Increasing the temperature further to 823 K shows more of the white particles. The first significant change that can be seen in these images is the appearance of a yellow phase at 873 K. According to the literature [4], there are two possible compounds of the involved elements constituting the sample which have this color; TeCl_4_ that has a pale-yellow color and yellow TeO_2_ (orthorhombic). Of these, only TeCl_4_ has a melting point and boiling point below 1000 K [3]. If these are formed something must happen to the sodium. Considering that these were done under oxidizing conditions, most likely the sodium is oxidized. This is possible according to the literature [15] as sodium is oxidized in air when heat is applied. Alternatively, the tellurium could have reacted with the sodium and formed a sodium-tellurium compound.

Sodium chloride has no phase change up to 1000 K that would change the appearance of it, according to the melting point (1074 K) and the boiling point (1686 K) [3]. Observing the result in Fig. 4 for the 1023 K temperature, nothing except the yellow phase remains. This is indicating that something has formed which has removed the sodium chloride or reacted with it, forming something more volatile than the sodium chloride.

Comparing the TGA result and the furnace experiments, during the first mass change of the sample (S2) in Fig. 3, something is slowly being volatilized between the temperatures 673 and 873 K. Observing the crucible at these temperatures in Fig. 4, this correlates to the increasing appearance of the white particles of the sample. Thus, a potential cause of the mass loss observed by the TGA can be correlated to the black part (tellurium) of the sample.

At the temperature 873 K in Fig. 3, the sample (S2) is still slowly losing mass. Correlating this to the result from the furnace experiment in Fig. 4, a yellow phase is being formed on the sample and the crucible. Moreover, the black part has decreased significantly. Thus, an explanation could be that the yellow phase is what is being volatilized as it covers the sample and the crucible.

Observing the two samples heated to 923 and 973 K, only a slight change can be seen as the sample goes from a mixture (or close to it) to an almost melted stage. Comparing this to the TGA result (S2) in Fig. 3, no drastic change occurs between 923 and 973 K. This is indicating that it is the same species that is continuously volatilized at these temperatures.

For the final two samples in Fig. 4, heated to temperatures of 1023 and 1073 K, the samples are almost completely consumed; only the yellow phase remains, forming a yellow glazed surface. The TGA result at the same temperature (S2) in Fig. 4, is showing a mass loss of the sample, the tellurium and the sodium chloride reference. However, the sodium chloride reference is only starting to lose mass whereas the other two are showing significant mass losses. Considering the boiling point of sodium chloride (1686 K [3]), there should not be a rapid loss of sodium chloride at these temperatures. Thus, this is indicative that something has interacted with the sodium chloride and formed a more volatile compound.

Thus, by comparing both TGA and the furnace results it is possible that the tellurium is affected by the sodium chloride, as the behavior of the latter is not consistent with what would be expected if no interaction occurred. However, some differences exist between the experiments. The main are the temperature gradient, sample amount, and sodium chloride particles size. These could have an impact on the results, mainly the particles size difference as the smaller particles have bigger total contact surface area than that of the bigger sized particles. Thus, interaction between the sodium chloride and tellurium is more likely during the TGA. The other two are relevant, but the TGA is limited by these two. Still, an effect is the temperature gradient is still possible. However, as the furnace experiments aim to investigate a specific temperature and not the heating to that temperature the temperature gradient needs to be as high as possible. Furthermore, the sample amounts were increased to ensure that something was observable afterward.

The experimental results for the inert conditions showed little difference between the reference and the samples. Observing the outcome from the two reference samples, it can be observed that at the temperatures were a mass loss is observed for the sodium chloride only minor amounts of tellurium remains in the corresponding reference. Thus, it is possible that most of the tellurium is gone by the time that something could occur with the sodium chloride. Therefore, it is not possible to completely exclude that nothing will happen.

A mass increase was observed for the tellurium reference case between 800–1000 K under oxidizing conditions. This was attributed to the tellurium-oxide formation. This increase was not present when sodium chloride was added to tellurium. Instead, a slight decrease was noticed. This could be explained by either sodium chloride physically preventing the oxidation of the tellurium in the crucible or the formation of a reaction product that is more volatile than tellurium oxide, or alternatively a reaction product that prevents the oxidation of tellurium. Moreover, the sodium chloride reference mass loss behavior was very similar in both atmospheres. Thus, any change of the samples (S1, S2, and S3) before the second main mass losses of the samples are only due to either the tellurium behavior or to the tellurium and sodium chloride interaction. This was supported by the observation in the furnace experiments, where the sodium chloride changes occurred at a lower temperature compared to the melting point.

It is not possible to give a definitive answer if a possible reaction occurred in the gas phase, liquid phase, solid phase or phase boundaries. However, based on the melting points and boiling points, the possible reaction observed most likely occurred in the liquid–liquid or liquid–solid phase. This would not exclude a gas–phase interaction, but this would not be observable in the TGA or furnace experiments, as anything volatilized is almost directly transported away from the sample. Thus, no gas reaction can be noticed by the scale or in the crucible.

## Conclusions

The results found in this study indicates that a dryout after using seawater for cooling could increase releases of tellurium under oxidizing conditions. As tellurium is entrapped by the cladding and requires oxidizing of the cladding to be released, such a scenario is feasible. Therefore, in the event of e.g. a dry-out of the core that occurs after using seawater, the consequences would be an increase in the tellurium source term.

For future studies, the next step would be to use a system capable of online measurement of the volatilized material, specifically speciation. Moreover, as tellurium has a low boiling point a setup using several furnaces could also be used to allow for preheating and consequently volatilization of the materials. The volatilized material would then be transported to a reaction zone, maintained at a higher temperature.
